# SIRT4-Catalyzed Deacetylation of Axin1 Modulates the Wnt/β-Catenin Signaling Pathway

**DOI:** 10.3389/fonc.2022.872444

**Published:** 2022-05-30

**Authors:** Yuting Wang, Jicheng Yue, Mingzhe Xiao, Xiaomei Lu, Yuen Eugene Chin

**Affiliations:** ^1^ Institutes of Biology and Medical Sciences, Soochow University School of Medicine, Suzhou, China; ^2^ The Affiliated Wuxi People’s Hospital of Nanjing Medical University, Wuxi, China; ^3^ Peninsula Cancer Research Center, Binzhou Medical University, Yantai, China; ^4^ Institute of Health Sciences, Shanghai Institutes for Biological Sciences, Chinese Academy of Sciences, Shanghai, China; ^5^ Cancer Hospital of Xinjiang Medical University, Urumqi, China

**Keywords:** Axin1, SIRT4, deacetylation, β-catenin, Wnt signaling pathway

## Abstract

Axin1 is a fundamental scaffolding protein of the destruction complex in the canonical Wnt signaling pathway, which plays a critical role in various biological processes. However, how Axin1 is regulated in the activation of the canonical Wnt signaling pathway remains elusive. Here, we report that Axin1 is constitutively acetylated in resting cells. Upon stimulation with Wnt, SIRT4 translocates from mitochondria to the cytoplasm and catalyzes Axin1 deacetylation, thus turning off the destruction complex. In this process, Lys147, a residue in the RGS domain of Axin1, plays a key role. We proved that the Axin1-K147R mutant impairs the assembly of β-TrCP to the destruction complex, which leads to β-catenin accumulation even without Wnt stimulation. In summary, our work proposes a new model for better understanding the initial stage of the canonical Wnt signaling pathway in which SIRT4 translocates from mitochondria into the cytoplasm to deacetylate Axin1-K147 after Wnt stimulation, which results in reduced assembly of β-TrCP to the destruction complex.

## Introduction

The Wnt/β-catenin signaling cascade plays a crucial role in regulating many biological processes, especially those involved in cell or organ development. Abnormal Wnt/β-catenin signaling often leads to cell or organ developmental failure or diseases (1–3). In the absence of Wnt ligands, cytosolic β-catenin is maintained at an extremely low level in the cytoplasm by finely regulated continuous synthesis and degradation machinery. β-catenin degradation takes place within a so-called destruction complex, which includes axis inhibition scaffold proteins (Axin1 and Axin2), adenomatous polyposis coli (APC), glycogen synthase kinase-3β (GSK3β), casein kinase-1 (CK-1), protein phosphatase 2A (PP2A) and E3-ubiquitin ligase β-TrCP (4, 5). During the degradation process, cytosolic β-catenin captured by the destruction complex is sequentially phosphorylated by CK-1 and GSK3β in a conserved Ser/Thr-rich sequence near the amino terminus, a process requiring scaffolding of the kinases and β-catenin by Axin (6). Phosphorylated β-catenin can therefore be recognized by β-TrCP and subjected to protein degradation through the ubiquitin–proteasome pathway (7, 8). In the Wnt-on state, Wnt ligands combine their receptor with Frizzled or LRP5/6 to recruit disheveled proteins to the cell membrane, ensuing the dissociation of the destruction complex and ultimately preventing β‐catenin degradation. Subsequently, β‐catenin accumulates in the cytoplasm and then translocates into the nucleus. In the nucleus, β-catenin is engaged in TCF/LEF transcription factors to regulate the Wnt signaling pathway (9).

In the canonical Wnt signaling pathway, the main function of Axin1 is to tether most components of the β-catenin destruction complex together (10). At least in frog embryos, it is the least abundant member of all components of the destruction complex (11). Thus, it is supposed that Axin1 is a rate-limiting protein in the destruction complex. When the amount of the rate-limiting protein Axin1 increases to some extent, the degradation of β-catenin mediated by the destruction complex rises greatly (12). In short, a small change in Axin1 is enough to regulate the degradation of β-catenin (13, 14). Therefore, we speculated that in addition to protein quantity, posttranslational modification (PTM) of Axin1 may be important to balance the cellular Wnt signaling response.

Acetylation is a kind of protein posttranslational modification that generally neutralizes the positive charges of lysine residues and regulates protein interactions within a hydrophobic complex (15, 16). PTM changes are usually the first steps of many signaling pathways. We analyzed the PTM of destruction complex components in the early stage of Wnt signaling and found that Axin1 deacetylation is critical for proper Wnt signal transduction. Generally, deacetylation is controlled by histone deacetylases (HDACs). HDACs include classical zinc-dependent HDACs and NAD^+^-dependent HDACs, which are also known as silent mating-type information regulator 2 homologs (sirtuins) (17). Sirtuins are highly conserved from bacteria to humans (18). Seven SIRT family members (SIRT1–7) have been identified in mammals. They have diverse subcellular localizations and functions (19). Among them, SIRT4 is a multifunctional protein with enzymatic activities such as deacetylation (20), ADP-ribosyl transfer (21), and lipoamidase (22) activity. Generally, SIRT4 is supposed to be localized in the mitochondria and is involved in regulating metabolism and metabolic homeostasis (23, 24). However, it was recently proved that a small amount of SIRT4 presents in both cytosol and nucleus (25, 26). Interestingly, our preliminary results showed that SIRT4 could translocate from mitochondria to the cytoplasm after Wnt stimulation.

Here, we demonstrated that deacetylation of Axin1 regulates the canonical Wnt/β-catenin signaling pathway. Deacetylation of Axin1 is mediated by SIRT4, which translocates from mitochondria to the cytoplasm in response to Wnt stimulation. We provided strong evidence that SIRT4-mediated deacetylation of Lys147 was one of the crucial reactions in activation of the canonical Wnt signaling pathway, which results in reduced assembly of β-TrCP to the destruction complex. As a consequence, β-TrCP-catalyzed ubiquitination and ubiquitin-proteasome-dependent degradation of β-catenin were significantly inhibited.

## Results

### Axin1 Is Deacetylated During Wnt Signaling Pathway Activation

**Figure 1:**
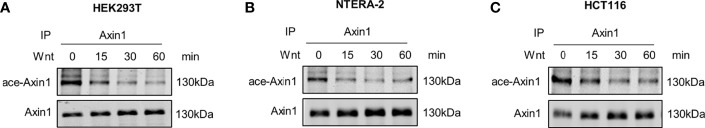
Axin1 is deacetylated during Wnt signaling pathway activation. **(A)** HEK293T (human embryonic kidney 293 cell), **(B)** NTERA-2 (pluripotent human embryonic carcinoma cell), **(C)** HCT116 (human colorectal carcinoma cell) cells were stimulated with Wnt3a-conditioned medium. Cell lysates at the indicated time points were collected and subjected to immunoprecipitation with anti-Axin1 antibody. The precipitated Axin1 protein was then subjected to SDS-PAGE followed by western blot analysis with the indicated antibodies.

Since Axin1 is considered to be a rate-limiting factor of the destruction complex (11), we sought to investigate whether dynamic acetylation of Axin1 occurs in the activation of the Wnt signaling pathway. To obtain enough Axin1 protein from HEK293T cells, a cell line with an intact Wnt signaling cascade and a high level of Wnt autocrine signaling (14), we pooled HEK293T cells from two 15 cm culture dishes. Axin1 was immunoprecipitated and analyzed by western blot with an anti-panacetylated lysine antibody. It was noted that acetylation of Axin1 was removed rapidly after Wnt3a stimulation (**Figure 1A**). We confirmed that deacetylation of Axin1 is widespread in the activation of the Wnt signaling pathway. Axin1 of NTERA-2, a pluripotent human embryonic carcinoma cell with an intact canonical Wnt/β-catenin cascade and a low level of Wnt autocrine signaling, and Axin1 of HCT116, a human colorectal carcinoma cell with a β-catenin mutation, were analyzed. Deacetylation of Axin1 was also observed after Wnt3a stimulation (**Figures 1B, C**).

### SIRT4 Translocates to the Cytoplasm to Regulate the Acetylation Levels of Axin1 and Promote β-Catenin Protein Accumulation

**Figure 2:**
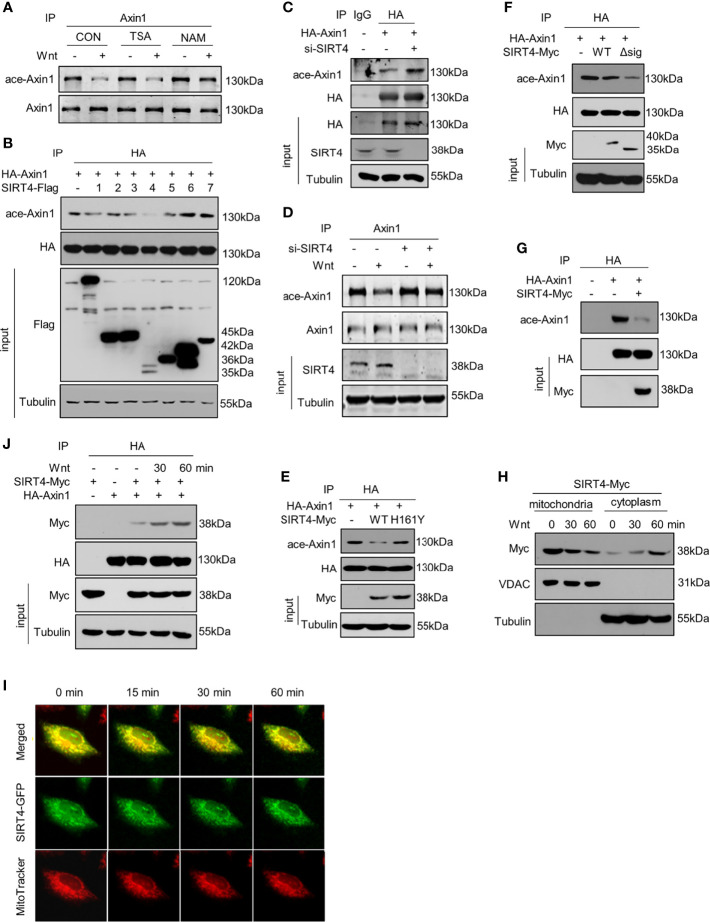
SIRT4 translocates to the cytoplasm and regulates the acetylation levels of Axin1 **(A)** HEK293T cells overexpressing HA-Axin1 were treated with the HDAC inhibitor TSA (2 μM) or SIRT inhibitor NAM (5 mM) for 2 hour, followed by treatment with Wnt3a-conditioned medium for 30 minutes. Stimulated lysates were subjected to immunoprecipitation using an Axin1 antibody and were subjected to SDS-PAGE followed by western blot analysis using the indicated antibodies. **(B)** HA-Axin1 with/without Flag-tagged sirtuins (1-7) were overexpressed in HEK293T cells, HA-Axin1 was immunoprecipitated, and the acetylation levels of Axin1 were analyzed by western blot. Tubulin protein levels were used as loading control. **(C)** HA-Axin1 with/without siSIRT4 RNA were overexpressed in HEK293T cells. HA-Axin1 was immunoprecipitated with anti-HA, and the acetylation levels of Axin1 were analyzed by western blot. Tubulin protein levels were used as loading control. **(D)** Immunoprecipitation analysis of Axin1 acetylation in HEK293T cells transfected with siSIRT4, followed by western blot analysis using the indicated antibodies after Wnt3a-conditioned medium stimulation. Tubulin protein levels were used as loading control. **(E)** HA-Axin1 was coexpressed with SIRT4-Myc or SIRT4-H161Y-Myc in HET293T cells for 48h. HA-Axin1 was immunoprecipitated with anti-HA, and the acetylation levels of Axin1 were analyzed by western blot. Tubulin protein levels were used as loading control. **(F)** HA-Axin1 was coexpressed with SIRT4-Myc or SIRT4-Δsig-Myc in HET293T cells for 48 h. HA-Axin1 was immunoprecipitated with anti-HA, and the acetylation levels of Axin1 were analyzed by western blot. Tubulin protein levels were used as loading control. **(G)** HA-Axin1 and SIRT4-Myc were overexpressed respectively in HET293T cells. The proteins were purified 48 h later, an *in vitro* deacetylation assay was performed and the acetylation levels of Axin1 were analyzed by western blot. **(H)** HEK293T cells transfected with SIRT4-Myc for 48 h were treated with Wnt3a-conditioned medium for indicated times and fractioned into cytoplasmic and mitochondrial fractions. The cell fractions were then analyzed by western blot with the indicated antibodies. VDAC was used as a loading control for mitochondria proteins. Tubulin was used as a loading control for cytoplasm proteins. **(I)** Hela cells were transfected with SIRT4-GFP, and 48 hours later, the cells were stained with MitoTrackerer™ Red CMXRos for 10 min and then treated with Wnt3a-conditioned medium for the indicated time. For the same scope, both green (SIRT4) and red (mitochondria) fluorescence were captured with a Leica Microsystem at the indicated time points after treatment with Wnt3a-conditioned medium. **(J)** HA-Axin1 was coexpressed with or without SIRT4-Myc in HET293T cells for 48 h. Some cells were treated with Wnt3a-conditioned medium as indicated. HA-Axin1 was immunoprecipitated with anti-HA, and the interaction between Axin1 and SIRT4 was detected by western blot.

We next sought to identify the enzyme that mediates the deacetylation of Axin1 and thus treated cells with the HDAC family inhibitor TSA or the SIRT family inhibitor NAM. It was noted that deacetylation of Axin1 was retarded by the SIRT family inhibitor NAM but not the HDAC family inhibitor TSA (**Figure 2A**). Therefore, we concluded that SIRT family members have an effect on this deacetylation process. However, it is not clear which SIRT member is responsible for Axin1 deacetylation in Wnt signaling pathway activation. To answer this question, we overexpressed seven members of the SIRT family and investigated their effects on Axin1 acetylation levels. Overexpression of SIRT4 significantly downregulated the acetylation level of Axin1 (**Figure 2B**). On the other hand, by knocking down endogenous SIRT4 with a specific siRNA, we noticed that the acetylation level of Axin1 was obviously upregulated (**Figure 2C**). Importantly, SIRT4 knockdown retarded Wnt3a-induced deacetylation of Axin1 (**Figure 2D**) similar to NAM treatment (**Figure 2A**). In addition, the inactivated mutant SIRT4-H161Y was co-overexpressed with Axin1 to confirm the deacetylation effect of SIRT4 on Axin1, SIRT4-H161Y lost the deacetylation effect on Axin1 compared with SIRT4-WT (**Figure 2E**). Normally, SIRT4 is located in mitochondria (21). To amplify SIRT4’s effect on Axin1 deacetylation, we constructed a mitochondrial targeting sequence deletion mutant SIRT4-Δsig, which lost the ability to enter mitochondria. Interestingly, when SIRT4 was retained in the cytoplasm more Axin1 was deacetylated (**Figure 2F**). Finally, we purified both Axin1 and SIRT4 proteins and performed an *in vitro* deacetylation assay. It was shown that SIRT4 could directly catalyze the deacetylation of Axin1 (**Figure 2G**).

Previous studies have found that Axin1 is a core component of the destruction complex, which is located in the cytoplasm, whereas SIRT4 is a protein located in mitochondria (21). We hypothesized that SIRT4 could translocate from mitochondria to the cytoplasm in response to Wnt stimulation. After treatment with Wnt3a-conditioned medium, we isolated the mitochondria and cytoplasm of HEK293T cells and detected SIRT4 protein levels. The results showed that SIRT4 decreased in the mitochondria and increased in the cytoplasm (**Figure 2H**). A small amount of SIRT4 enriched in the cytoplasmic fraction before Wnt stimulation supports the report that a tiny amount of SIRT4 is located in the cytosol (25). To further confirm this, with the help of a SIRT4-GFP fusion protein, we observed distinct separation of green (SIRT4-GFP) and red (MitoTracker) fluorescence, indicating that SIRT4 was translocated from mitochondria to the cytoplasm in a time-dependent manner after Wnt3a stimulation (**Figure 2I**). In addition, we noticed that Wnt treatment increased the interaction between SIRT4 and Axin1 in a time-dependent manner (**Figure 2J**). Taken together, our results confirm that Wnt3a regulates the subcellular localization of SIRT4, which catalyzes the deacetylation of Axin1.

**Figure 3:**
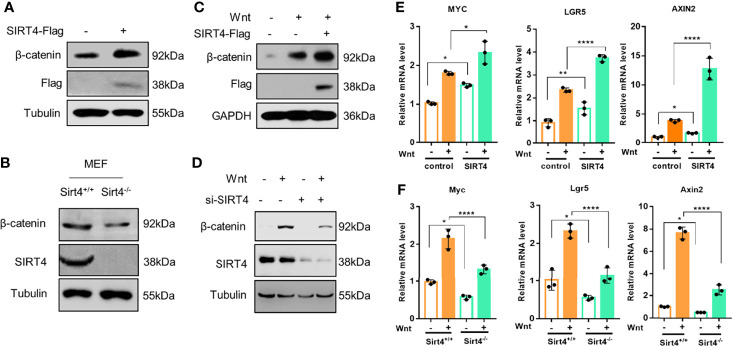
SIRT4 positively regulates the Wnt/β-catenin signaling pathway. **(A)** HEK293T cells were transfected with EV and SIRT4-Flag respectively, the cells were collected and the whole cell lysates were analyzed by western blot with the indicated antibodies. **(B)** Sirt4^+/+^ MEFs and Sirt4^−/−^ MEFs were prepared from WT and Sirt4 knockout mice respectively and maintained in DMEM with 10% FBS. Whole cell lysates were analyzed by western blot with the indicated antibodies. **(C)** HEK293T cells were transfected with EV or SIRT4-Flag, the cells were treated with Wnt3a-conditioned medium for 30 min. The whole cell lysates were analyzed by western blot with the indicated antibodies. **(D)** HEK293T cells were transfected with/without siSIRT4 for 48 h, then the cells were treated with Wnt3a-conditioned medium for 30 min. The whole cell lysates were analyzed by western blot with the indicated antibodies. **(E)** HEK293T cells were transfected with EV and SIRT4-Flag respectively, the cells were treated with Wnt3a-conditioned medium for 3 h. Total RNA was extracted with TRIzol, and the mRNA levels of β-catenin target genes (AXIN2, LGR5 and MYC) were analyzed with RT-qPCR (n = 3; error bars represent ± SD, *p < 0.05, **p < 0.01, ****p < 0.0001; two-way ANOVA with multiple comparisons followed by Bonferroni *post hoc* test). **(F)** Sirt4^+/+^ MEFs and Sirt4^−/−^ MEFs were stimulated with Wnt3a for 3 h. Total RNA was extracted with TRIzol, and the mRNA levels of β-catenin target genes (Axin2, Lgr5 and Myc) were analyzed with RT-qPCR (n = 3; error bars represent ± SD, *p<0.05, **** p < 0.0001; two-way ANOVA with multiple comparisons followed by Bonferroni *post hoc* test).

Given that the deacetylation of Axin1 is SIRT4 dependent, we speculated that SIRT4 may affect the activation of the canonical Wnt signaling pathway. Then, we explored the effect of SIRT4 on the Wnt signaling pathway. β-catenin is the key effector responsible for transduction of the Wnt signaling pathway to the nucleus (4). The accumulation of cytoplasmic β-catenin is the key switch in the canonical Wnt pathway (4). To study the effect of SIRT4 on the Wnt signaling pathway, we detected the level of β-catenin. Interestingly, overexpression of SIRT4 upregulated cellular β-catenin protein levels (**Figure 3A**). In contrast, SIRT4 knockout robustly downregulated β-catenin protein levels in MEFs (**Figure 3B**). Furthermore, overexpression of SIRT4 increased Wnt3a stimulation-induced β-catenin accumulation (**Figure 3C**). When cells were transfected with siSIRT4, we found that knockdown of endogenous SIRT4 significantly inhibited Wnt3a-induced β-catenin accumulation (**Figure 3D**). Correspondingly, the transcription of the target genes of canonical Wnt signaling was enhanced by SIRT4 overexpression (**Figure 3E**), while Sirt4 knockout inhibited it (**Figure 3F**). These results indicated that SIRT4 plays an important role in promoting canonical Wnt signaling pathway activation. However, we were not sure whether SIRT4-mediated deacetylation of Axin1 plays a key role in this process.

### SIRT4-Regulated Deacetylation of the Axin1-K147 Residue Is Crucial for the Activation of the Wnt/β-Catenin Signaling Pathway

**Figure 4:**
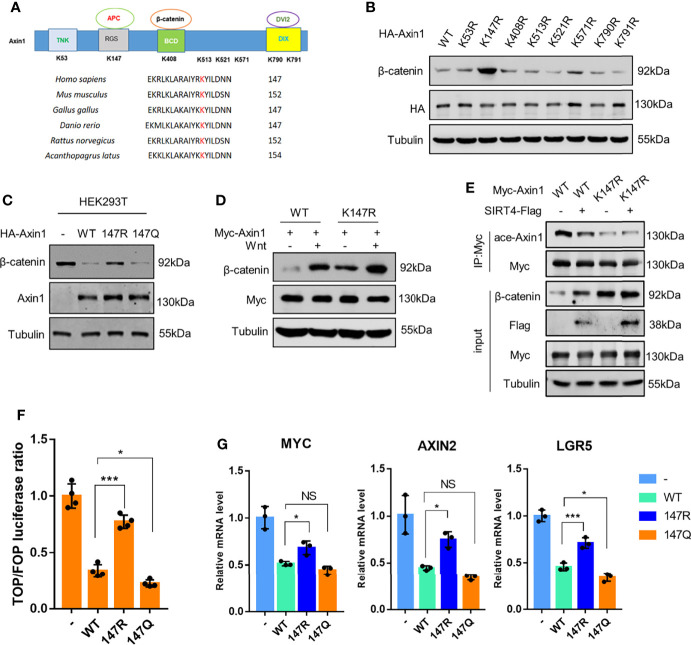
SIRT4-regulated deacetylation of the Axin1-K147 residue is crucial for the activation of the canonical Wnt/β-catenin signaling pathway. **(A)** Mass spectrometry analysis of the potential acetylation sites of Axin1. Multiple sequence alignment among species showed a conserved Lys147 residue in Axin1. **(B)** HA-Axin1 mutants (K53R, K147R, K408R, K513R, K521R, K571R, K790R, K791R) were overexpressed in HEK293T cells respectively, the whole cell lysates were analyzed by western blot with the indicated antibodies. **(C)** HEK293T cells were transfected with HA-Axin1-WT, HA-Axin1-K147R or HA-Axin1-K147Q. The whole cell lysates were analyzed by western blot with the indicated antibodies. **(D)** HEK293T cells were transfected with Myc-Axin1-WT or Myc-Axin1-K147R and then treated with Wnt3a conditioned medium for 30 min. The whole cell lysates were analyzed by western blot with the indicated antibodies. **(E)** HEK293T cells were cotransfected with Myc-Axin1/Myc-Axin1-K147R and SIRT4-Flag as indicated. The whole cell lysates were collected and analyzed by western blot with the indicated antibodies as input, Myc-Axin1 was immunoprecipitated with anti-Myc and the acetylation levels were analyzed by western blot. **(F)** Dual-luciferase reporter assay of TOP/FOP activity in HEK293T cells transfected with HA-Axin1-WT, HA-Axin1-K147R or HA-Axin1-K147Q (n=4; error bars represent ± SD, *p<0.05, ***P<0.0001; ordinary one-way ANOVA with multiple comparisons followed by Holm-Sidak’s test). **(G)** HEK293T cells were transfected with HA-Axin1-WT, HA-Axin1-K147R and HA-Axin1-K147Q respectively. Total RNA was extracted with TRIzol and the mRNA levels of β-catenin target genes (AXIN2, LGR5 and MYC) were analyzed with RT-qPCR (n = 3; error bars represent ± SD, *p<0.05, ***p<0.001; n=0.3>0.05 NS, no significant; ordinary one-way ANOVA with multiple comparisons followed by Holm-Sidak’s test).

We confirmed that SIRT4-mediated deacetylation of Axin1 is a crucial step in β-catenin accumulation. Human Axin1 was purified and subjected to mass spectrometric analysis, and eight lysine residues with acetylation were detected: Lys53, Lys147, Lys408, Lys513, Lys521, Lys571, Lys790 and Lys791, which are distributed in different domains of Axin1 (**Figure 4A**). Mutants of Axin1 (K53R, K147R, K408R, K513R, K521R, K571R, K790R or K791R), whose lysine at positions 53, 147, 408, 513, 521, 571, 790 or 791 was replaced with arginine to mimic the nonacetylated state of the lysine residue, were prepared. Then, we transfected HEK293T cells with vectors encoding wild-type or mutant Axin1 to determine the effect of the change on the biological function of Axin1. Our results revealed that the K147R Axin1 mutant dramatically increased β-catenin protein levels (**Figure 4B**), implying that Axin1 could not assemble functional destruction complexes if Lys147 could not be acetylated. Next, we constructed another mutant of Lys147 (K147Q). This mutant had a replacement of the lysine at position 147 with glutamine to mimic the acetylated state of the lysine residue. Wild-type Axin1 and the K147Q mutant obviously decreased the β-catenin amount, whereas similar amounts of the K147R mutant lost the property of wild-type Axin1 to some extent (**Figure 4C**). We transfected HEK293T cells with Axin1-WT and Axin1-K147R and then treated them with Wnt3a. Our results showed that in the Axin1-WT group, intracellular β-catenin significantly accumulated after Wnt3a stimulation. While overexpression of Axin1-K147R significantly upregulated β-catenin protein levels compared with Axin1-WT and only slightly upregulated the level of β-catenin protein in the Axin1-K147R group when stimulated by Wnt3a (**Figure 4D**). This result suggested that Axin1-K147R significantly obstructs Wnt-regulated β-catenin accumulation. However, although Axin1-K147R significantly inhibited Wnt-regulated β-catenin accumulation, β-catenin protein was still slightly upregulated. We hypothesized that Wnt may regulate endogenous Axin1 deacetylation and further promote β-catenin accumulation. These data suggested that the acetylation level of the Axin1-K147 residue was involved in the regulation of Axin1 function. In addition, sequence alignment revealed that Axin1-K147 was a highly conserved residue among different species (**Figure 4A**).

Although Axin1-K147 is a key residue in the regulation of Axin1 function, whether deacetylation of Axin1-K147 is a major contributor to SIRT4-mediated Axin1 deacetylation has still not been confirmed. We then coexpressed SIRT4 and Axin1 in HEK293T cells. The acetylation level of the K147R mutant decreased significantly compared to that of the wild type (**Figure 4E**, lane 1 vs lane 3). While the acetylation level of Axin1-WT was decreased by SIRT4 (**Figure 4E**, lane 1 vs lane 2), the acetylation level of Axin1-K147R was not affected by SIRT4 overexpression (**Figure 4E**, lane 3 vs lane 4), indicating that SIRT4-mediated deacetylation of Axin1 mainly occurred at Lys147. Consistently, while the β-catenin level in the Axin1-WT group was increased significantly by SIRT4, further β-catenin accumulation in the Axin1-K147R group was abolished (**Figure 4E**, panel 4).

Consistently, while Axin1-WT and the Axin1-K147Q mutant decreased the TOP/FOP flash ratio and the transcription levels of β-catenin target genes dramatically, the Axin1-K147R mutant failed to induce such events (**Figures 4F, G**). Taken together, these results implied that SIRT4-mediated deacetylation of the Axin1-K147 residue is a crucial step in the activation of the canonical Wnt/β-catenin signaling pathway.

### SIRT4-Catalyzed Deacetylation of Axin1-K147 Reduces the Accessibility of β-TrCP to the Destruction Complex

**Figure 5:**
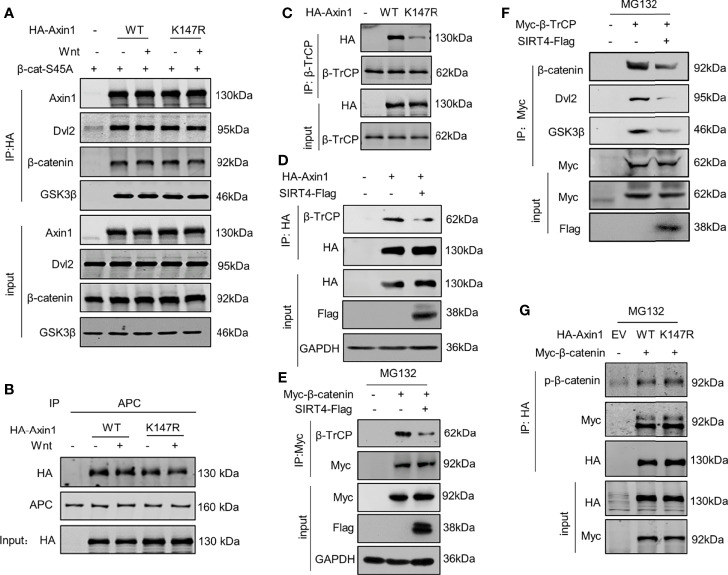
SIRT4-catalyzed deacetylation of Axin1-K147 reduces accessibility of β-TrCP to the destruction complex. **(A)** Both WT and K147R mutant HA-tagged Axin1 were cotransfected with 6×Myc-β-catenin-S45A. Cells were treated with Wnt3a-conditioned medium as indicated for 30 minutes before samples were collected. HA-Axin1 was immunoprecipitated with anti-HA beads. Immunoprecipitated proteins were analyzed by western blotting with the indicated antibodies. **(B)** HA-Axin1-WT or HA-Axin1-K147R were transfected in HEK293T cells as indicated. The cells were treated with Wnt3a-conditioned medium for 30 minutes and then were collected and subjected to coimmunoprecipitation with anti-APC. Axin1 and APC were detected by western blot with anti-HA or anti-APC antibody respectively. **(C)** HEK293T cells were transfected with HA-Axin1-WT/K147R respectively, whole cell lysates were collected, β-TrCP was immunoprecipitated with anti-β-TrCP. Both whole cell lysates and precipitates were analyzed by western blot with the indicated antibodies. **(D)** HEK293T cells were cotransfected with HA-Axin1 and SIRT4-Flag as indicated, whole cell lysates were collected, HA-Axin1 was immunoprecipitated with anti-HA. Both whole cell lysates and precipitates were analyzed by western blot with the indicated antibodies. **(E)** HEK293T cells were cotransfected with Myc-β-catenin and SIRT4-Flag as indicated, and then treated with 10 mM MG132 for 4 h, the whole cell lysates were collected, Myc-β-catenin was immunoprecipitated with anti-Myc. Both whole cell lysates and precipitates were analyzed by western blot with the indicated antibodies. **(F)** HEK293T cells were cotransfected with Myc-β-TrCP and SIRT4-Flag as indicated, and then treated with 10 mM MG132 for 4 h, the whole cell lysates were collected, Myc-β-TrCP was immunoprecipitated with anti-Myc. Both whole cell lysates and precipitates were analyzed by western blot with indicated antibodies. **(G)** HEK293T cells were cotransfected with Myc-β-catenin and HA-Axin1 (WT or K147R) as indicated with treated 10 mM MG132 for 4 h, and then whole cell lysates were collected, HA-Axin1 was immunoprecipitated with anti-HA. Both whole cell lysates and precipitates were analyzed by western blot with indicated antibodies.

The results above suggest that SIRT4 promotes β-catenin accumulation by regulating Axin1 acetylation. Next, we further explored the mechanism by which SIRT4 regulates β-catenin protein accumulation. Axin1 is a rate-limiting protein in the destruction complex, and it is commonly thought of as a scaffold that supports the stable presence of the destruction complex. Coimmunoprecipitation (co-IP) with HA-Axin1 showed that most components of the destruction complex were precipitated (**Figure 5A**). Intriguingly, although Lys147 is located in the RGS domain, a domain interacts with the SAMP domain of APC, and Axin1-K147R mutation did not damage the Axin1-APC interaction (**Figure 5B**). However, when the same amount of β-TrCP was immunoprecipitated, the Axin1-K147R mutant significantly reduced its interaction with β-TrCP compared with Axin1-WT (**Figure 5C**). In addition, overexpression of SIRT4 also reduced the interaction between Axin1 and β-TrCP (**Figure 5D**). Consistently, SIRT4 overexpression also decreased the protein level of β-TrCP precipitated with β-catenin (**Figure 5E**). Furthermore, SIRT4 overexpression reduced the interaction between β-TrCP and several components of the destruction complex (β-catenin, Dvl2 and GSK3β) (**Figure 5F**). Taken together, our findings demonstrated that SIRT4-mediated deacetylation of Axin1-K147 would result in reduced assembly of β-TrCP to the destruction complex.

Conventionally, β-TrCP is recruited by phosphorylated β-catenin; however, the Axin1-K147R mutation did not affect the assembly of GSK3β to the destruction complex (**Figure 5A**), and the levels of phosphorylated β-catenin in the destruction complex were not impaired by the K147R mutation (**Figure 5G**). We are not sure how deacetylation of Axin1-K147 can impair the interaction between β-TrCP and phosphorylated β-catenin.

### SIRT4 Downregulates the Ubiquitination of β-Catenin

**Figure 6:**
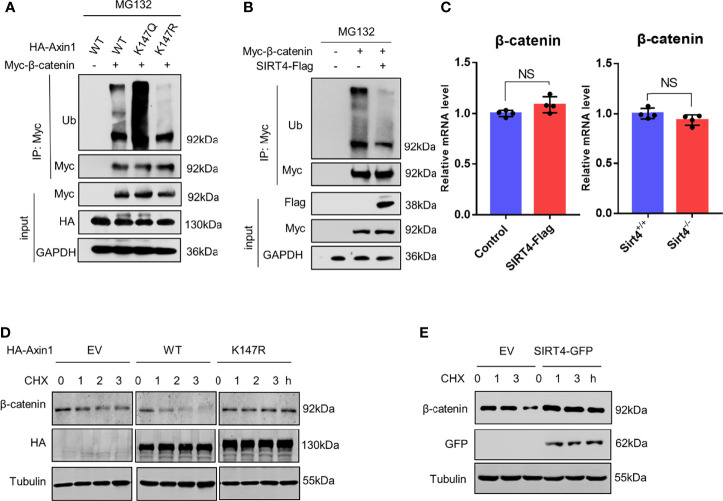
SIRT4 downregulates the ubiquitination of β-catenin. **(A)** HEK293T cells were cotransfected with Myc-β-catenin and HA-Axin1 (WT, K147Q or K147R) as indicated, the cells were treated with 10 mM MG132 for 4 h, and then whole cell lysates were collected, Myc-β-catenin was immunoprecipitated with anti-Myc. Both whole cell lysates and precipitates were analyzed by western blot with the indicated antibodies. **(B)** HEK293T cells were cotransfected with Myc-β-catenin and SIRT4-Flag as indicated, the cells were treated with 10 mM MG132 for 4 h, and then whole cell lysates were collected, Myc-β-catenin was immunoprecipitated with anti-Myc. Both whole cell lysates and precipitates were analyzed by western blot with the indicated antibodies. **(C)** RT–qPCR analysis of the mRNA of β-catenin (CTNNB) in HEK293T cells transfected with SIRT4-Flag. RT–qPCR analysis of the mRNA of β-catenin (CTNNB) in Sirt4^+/+^ MEFs or Sirt4^−/−^ MEFs (n=4, error bars represent ± SD, p=0.08>0.05 in HEK293T cells, p=0.1>0.05 in MEFs cells. NS-not significant, unpaired, two-tailed, parametric t-test were used). **(D)** HEK293T cells were transfected with HA-Axin1 (WT or K147R) as indicated, the cells were treated with 1 μM cycloheximide (CHX) for the indicated times. Whole cell lysates were collected and analyzed by western blot with the indicated antibodies. **(E)** HEK293T cells were transfected with or without SIRT4-GFP as indicated, the cells were treated with 1 μM cycloheximide (CHX) for the indicated times. Whole cell lysates were collected and analyzed by western blot with indicated antibodies.

Due to the decreased accessibility of β-TrCP to the destruction complex, we found that the levels of polyubiquitinated β-catenin were changed significantly. Compared to Axin1-WT, the levels of the polyubiquitinated β-catenin were inhibited by the Axin1-K147R mutation, whereas they were robustly increased by the K147Q mutation (**Figure 6A**). In line with our hypothesis, we found that SIRT4 overexpression dramatically inhibited the levels of polyubiquitinated β-catenin (**Figure 6B**).

Therefore, we concluded that SIRT4-mediated Axin1 deacetylation promotes β-catenin accumulation by reducing the levels of polyubiquitinated β-catenin and reducing its degradation speed. We confirmed that the expression of β-catenin was not promoted (**Figure 6C**). When protein translation was inhibited by CHX (cycloheximide), compared to the EV group, we found that the degradation speed of β-catenin was increased significantly because of the overexpression of Axin1-WT, but conversely, no perceptible change was observed when Axin1-K147R was overexpressed (**Figure 6D**). Similarly, SIRT4 overexpression also decreased the degradation speed of β-catenin (**Figure 6E**). In conclusion, SIRT4-mediated deacetylation of Axin1-K147 promotes β-catenin accumulation by reducing the ubiquitin-dependent degradation of β-catenin.

## Discussion

The Wnt/β-catenin signaling pathway plays a critical role in maintaining the self-renewal growth of embryonic stem cells and the development of various organs (27). In the regulation of the canonical Wnt signaling pathway, several components undergo numerous posttranslational modifications. One of the most well established is the sequential phosphorylation of the N-terminus of β-catenin in the destruction complex, which is required for β-TrCP-mediated ubiquitination (5). Acetylation of the canonical Wnt signaling pathway has also been intensively researched. (28–32). Distinct from the phosphorylation of β-catenin, acetylation of β-catenin occurs and functions outside of the destruction complex. In addition, acetylation of GSK3β, one component of the destruction component, was reported to reduce kinase activity and thus enhance β-catenin accumulation. In contrast, SIRT2-mediated deacetylation of GSK3β increases the phosphorylation level of β-catenin, which results in β-catenin degradation (33). In summary, acetylation of both GSK3β and β-catenin promotes Wnt/β-catenin pathway activation. However, the features of Axin1 acetylation are quite different. Deacetylation of Axin1 occurs in the destruction complex, which is dependent on Wnt stimulation. Even though several sirtuin proteins are involved in the Wnt signaling pathway, SIRT4 seems to be the only member that responds rapidly to Wnt stimulation and accumulates in the cytosol. Our work supports the report that SIRT4 is present within both the cytosol and nucleus (25, 26). However, how SIRT4 translocates from mitochondria to the cytoplasm and interacts with the destruction complex after Wnt stimulation remains elusive. In addition to direct deacetylation, SIRT1 positively regulates the translation of Dishevelled proteins in several cell lines and thus changes the gene expression of classic Wnt/Dvl target genes (34). As the SIRT1 and Dvl complex, it is reasonable to infer that some components of the destruction complex were substrate of SIRT1.

Several acetylated lysine residues were identified on Axin1, and it is reasonable to speculate that the residue located in a functional domain of Axin1 is crucial for its function. Unexpectedly, when Lys147 was mutated, the Axin1-APC interaction seemed to remain intact; however, the abundance of β-TrCP in the destruction complex was reduced. It was reported that upon Wnt treatment, β-catenin is released from the destruction complex after a while disassociation of the destruction complex and GSK3β sequestration could then occur together with Axin1 degradation, which usually takes hours to be observed (13, 14, 35), whereas deacetylation of Axin1 is an early event within 30 minutes at the initial stage.

## Materials and Methods

### Cell Culture and Transfection

HEK293T, NTERA-2, and HCT116 cells were obtained from the American Type Culture Collection, and L-Wnt3a-producing cells were a kind gift from Pro. Xiaoren Zhang. HEK293T, NTERA-2 and L-Wnt-3a cells were cultured in DMEM (high glucose) (HyClone) supplemented with 10% fetal bovine serum (FBS) (Gemini), 100 units/ml penicillin and 100 μg/ml streptomycin (Sangon Biotech, Shanghai). HCT116 cells were cultured in RPMI-1640 medium (HyClone) supplemented with 10% FBS, 100 units/ml penicillin and 100 μg/ml streptomycin. Cell transfection was carried out with Lipofectamine 2000 transfection reagent following the manufacturer’s instructions for Invitrogen (Thermo Fisher Scientific, Waltham, MA). For siRNA transfection, cells were seeded in 6-well plates (1 × 10^6^ cells/well) and incubated for 48 h at 37°C with 5% CO_2_. Then the cells were transfected with siRNA using siRNA mate reagent (GenePharma) after reaching 70% confluence.

### Plasmid Construct

SIRT4-Flag was cloned into the 5’ FLAG-pcDNA 3.0 vector. Human or mouse Axin1 was cloned into 5’FLAG-pCS2. SIRT4-Myc and SIRT4-Δsig-Myc were cloned into the Myc-N1 vector. K53R, K147R, K408R, K513R, K521R, K571R, K790R, K791R and K147Q mutants of Axin1 and H161Y mutant of SIRT4 were generated according to the site-directed mutagenesis protocol of Stratagene, Agilent Technologies Inc. (Santa Clara, CA). Human β-catenin was cloned into a 5’6xmyc-pcDNA 3.0 vector and introduced according to the site-directed mutagenesis protocol. All constructs were confirmed by Sanger sequencing analysis.

### Antibodies and Reagents

The following primary antibodies were commercially obtained: anti-acetylated-lysine antibody (Cell Signaling Technology, 9441); anti-SIRT4 antibody (Abcam, ab10140); anti-β-catenin antibody (Cell Signaling Technology, 9562); anti-HA antibody (Santa Cruz, sc-7392); anti-Myc antibody (Santa Cruz, sc-40); anti-Flag antibody (Sigma, F1804); anti-tubulin antibody (Sigma, T619); anti-phospho-S33/37/T41 β-catenin antibody (Cell Signaling Technology, 9561); anti-β-TrCP antibody (Cell Signaling Technology, 4394); anti-β-TrCP antibody (Cell Signaling Technology, 11984) and anti-dishevelled 2 antibody (Abcam, ab228804); anti-GSK3β antibody (Cell Signaling Technology, 12456); anti-APC antibody (Abcam, ab40778). FLAG peptide was purchased from Sigma–Aldrich (St. Louis, MO). Small interfering RNAs (siRNAs) against SIRT family mRNAs were purchased from Santa Cruz Biotechnology, Inc. (SIRT1 siRNA (h), sc-40986, SIRT3 siRNA (h), sc-61555, SIRT4 siRNA (h), sc-63024, SIRT5 siRNA (h), sc-63026, SIRT6 siRNA (h), sc-63028, SIRT7 siRNA (h), sc-63030). Chemicals, including NAM (A2984) and TSA (T1952), were obtained from Sigma. Wnt3a-conditioned medium was derived from stably transfected L-Wnt3a cells.

### Luciferase Reporter Assay

HEK293T cells were seeded in 24-well plates and transfected with 5 ng TK-Renilla and 30 ng TOP-flash or FOP-flash reporter constructs. Cells were treated with Wnt3a-conditioned medium for 12 h and lysed with passive lysis buffer. Luciferase activity was measured using a Dual Luciferase Reporter Assay System (Promega Corporation, E1960) according to the manufacturer’s instructions.

### Immunoprecipitation (IP), Coimmunoprecipitation (co-IP) and Pull-Down Assay

Cells were washed with PBS and collected at 3000 rpm for 5 min at 4°C. For co-IP and IP, cells were lysed with co-IP buffer (50 mM Tris pH 8.0, 0.5% NP-40, 1 mM EDTA, 150 mM NaCl) or RIPA on ice and clarified at 12000 rpm for 10 min at 4°C. The lysate was incubated with primary antibody for 2-4 hours at 4°C, followed by the addition of a 50% slurry of protein A/G magnetic beads (B23202, Bimake) and incubation overnight at 4°C or direct incubation with antibody-conjugated beads overnight at 4°C. Flag-tagged proteins were purified by anti-FLAG M2 affinity gel (A2220, Sigma) following the manufacturer’s instructions. A peptide pulldown assay was performed in buffer PD (20 mM HEPES, pH 7.9, 20% v/v glycerol, 0.2 mM EDTA, 0.2% Triton X‐100, 2 mM DTT). The western blots were scanned and analyzed on a LI-COR system (Odyssey).

### Mass Spectrometry Analysis

Flag-tagged Axin1 was transfected with Lipofectamine 2000 into HEK293T cells, which were cultured in 10-cm Petri dishes. After 48 hours, cells from two dishes were collected to immunoprecipitate Axin1. The immunoprecipitated Axin1 was then separated by SDS–PAGE. Peptides were extracted and analyzed with a Thermo LC–MS/MS system.

### RNA Isolation and Quantitative RT–PCR

RNA was extracted from cells with TRIzol reagent (Invitrogen), and cDNA was prepared with a PrimeScript RT Reagent kit (Takara) according to the manufacturer’s instructions. Real-time PCR (MYC: GGCTCCTGGCAAAAGGTCA, CTGCGTAGTTGTGCTGATGT, LGR6: AGCCCTGT GAGTACCTCTTTG, CAGCACCAGTCCATTGCAGA, AXIN2: TACACTCCTTATTGGGCGA TCA, TTGGCTACTCGTAAAGTTTTGGT, CTNNB1: CATCTACACAGTTTGATGCTGCT, GCAGTTTTGTCAGTTCAGGGA, Myc: ATGCCCCTCAACGTGAACTTC, CGCAACATAGG ATGGAGAGCA, Lgr6: GAGGACGGCATCATGCTGTC, GCTCCGTGAGGTTGTTCATACT, Axin2: TGACTCTCCTTCCAGATCCCA, TGCCCACACTAGGCTGACA) was performed with SYBR Green Master Mix (YEASEN) on an ABI QuantStudio (Applied Biosystems). Relative quantitation of gene expression was calculated with the ΔΔCt method.

### *In Vitro* Deacetylation Assay

HA-Axin1 and SIRT4-Myc were overexpressed respectively in HET293T cells. The proteins were purified, and an *in vitro* deacetylation assay was performed as previously described (36).

### Cytosolic β-Catenin and Mitochondrial Fraction Preparation

To collect cytosolic proteins from mammalian cells, cells were collected and washed with PBS and incubated in fraction buffer (10 mM KCl, 10 mM Tris pH 7.5, 2 mM EDTA with PMSF and protease inhibitor added) on ice for 30 min. The cells were then stroke 30 times with a 1 mL syringe needle and centrifuged at 12000 rpm for 40 min at 4°C. The supernatant was collected as the cytosolic fraction containing cytosolic β-catenin. Mitochondria were extracted using a Mitochondria Isolation Kit (Sigma) following the manufacturer’s protocol. In brief, 2 ×10^7^ cells were pelleted by centrifuging the harvested cell suspension, and then mitochondria isolation reagent was added to the cell pellets. The cell resuspension was centrifuged at 700 × g for 10 min at 4°C, and then the supernatant was transferred to a new 1.5 ml tube and centrifuged at 12,000 × g for 15 min at 4°C. The supernatant (cytosolic fraction) was transferred to a new tube, and the pellet contained the isolated mitochondria. The pellet was further lysed to yield the final mitochondrial lysate. The extracted proteins were prepared for subsequent western blotting analysis.

### Statistical Analysis

All data are presented as the means ± SD. For single comparisons, an unpaired two-tailed Student’s t tests were used, p< 0.05 was considered statistically significant, NS indicate no significant. Statistical significance is indicated by asterisks (* p < 0.05, ** p < 0.01, *** p < 0.001). For multiple groups comparisons, one-way ANOVA Holm-Sidak’s test were used and two-way ANOVAs with Bonferroni *post hoc* test for assessing the combination of SIRT4 overexpression/knockout and Wnt treatment. The Statistical analyses were performed using the GraphPad Prism 7.0 and SPSS 23.0 software package.

## Data Availability Statement

The original contributions presented in the study are included in the article/**Supplementary Material**. Further inquiries can be directed to the corresponding authors.

## Author Contributions

The conceptualization and supervision were carried out by YC and XL. The investigation was carried out by YW and MX, and these authors contributed equally. YW and JY provided critical intellectual input for experimental design and prepared the manuscript. All authors contributed to the article and approved the submitted version.

## Funding

This research was supported by the National Natural Science Foundation of China (Grant Nos. 82030077, 81530083, 81820108023, 31700789) and the National Key Research and Development Project (2016YFC1302402, U1603284, 2018YFC1705505).

## Conflict of Interest

The authors declare that the research was conducted in the absence of any commercial or financial relationships that could be construed as a potential conflict of interest.

## Publisher’s Note

All claims expressed in this article are solely those of the authors and do not necessarily represent those of their affiliated organizations, or those of the publisher, the editors and the reviewers. Any product that may be evaluated in this article, or claim that may be made by its manufacturer, is not guaranteed or endorsed by the publisher.
