# Supplementary figures and images for: SIRT4-Catalyzed Deacetylation of Axin1 Modulates the Wnt/β-Catenin Signaling Pathway
Source: Front Oncol. 2022 May 30;12:872444. doi: 10.3389/fonc.2022.872444 (PMC9190513; doi:10.3389/fonc.2022.872444)

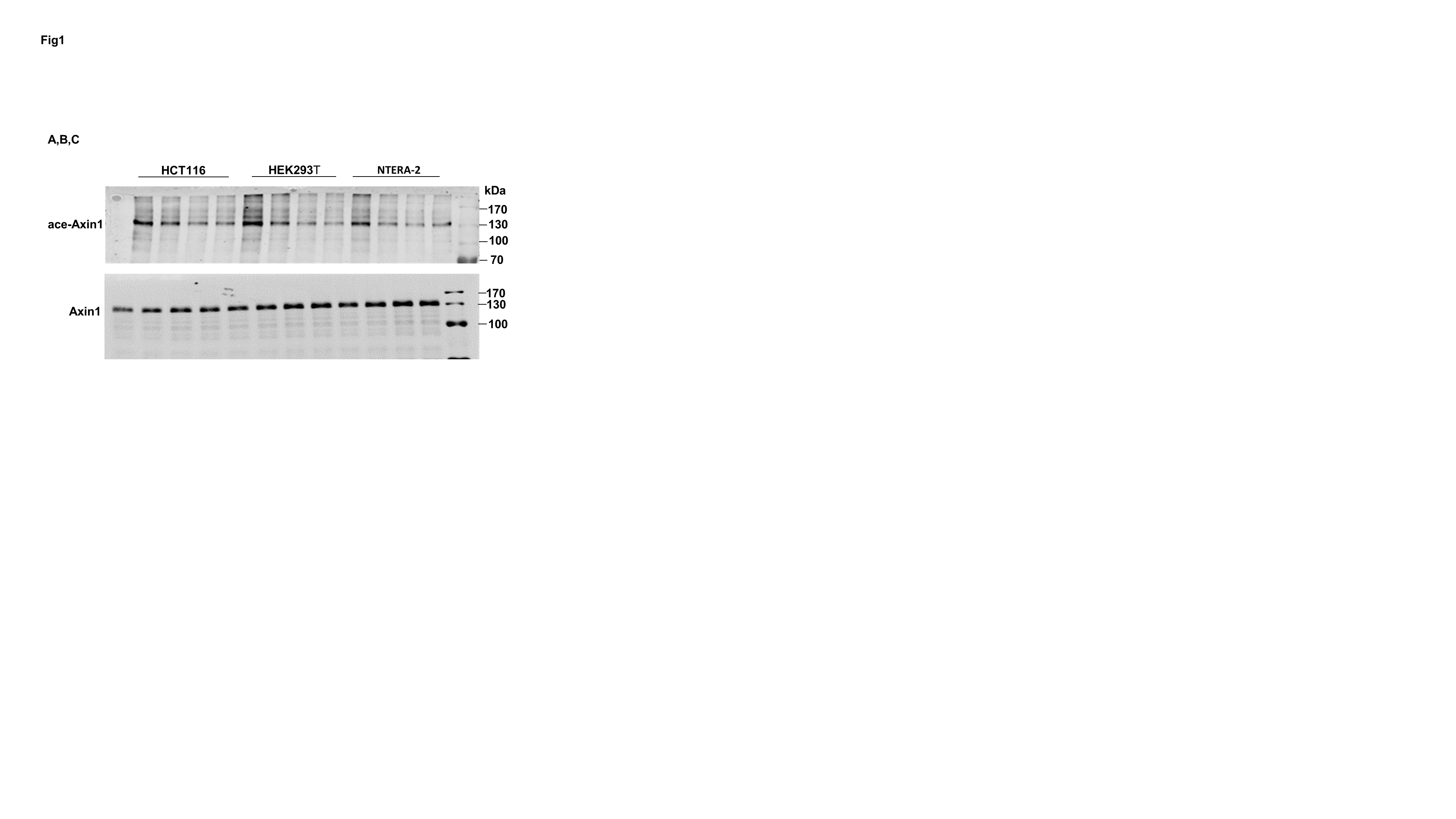

Supplement: Supplementary file 1 [file DataSheet_1.zip › 2022-5-1/幻灯片1.TIF]

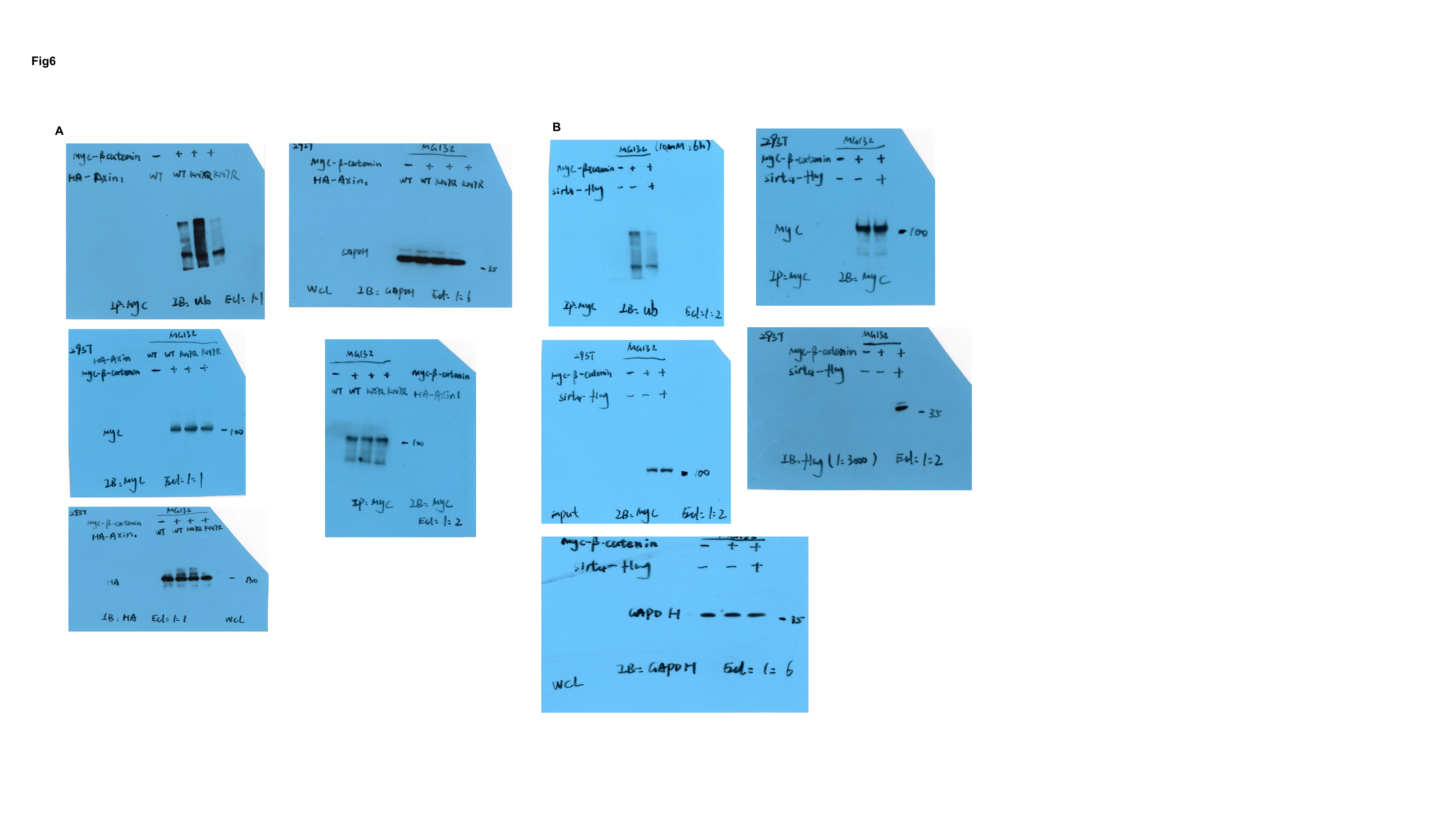

Supplement: Supplementary file 1 [file DataSheet_1.zip › 2022-5-1/幻灯片10.TIF]

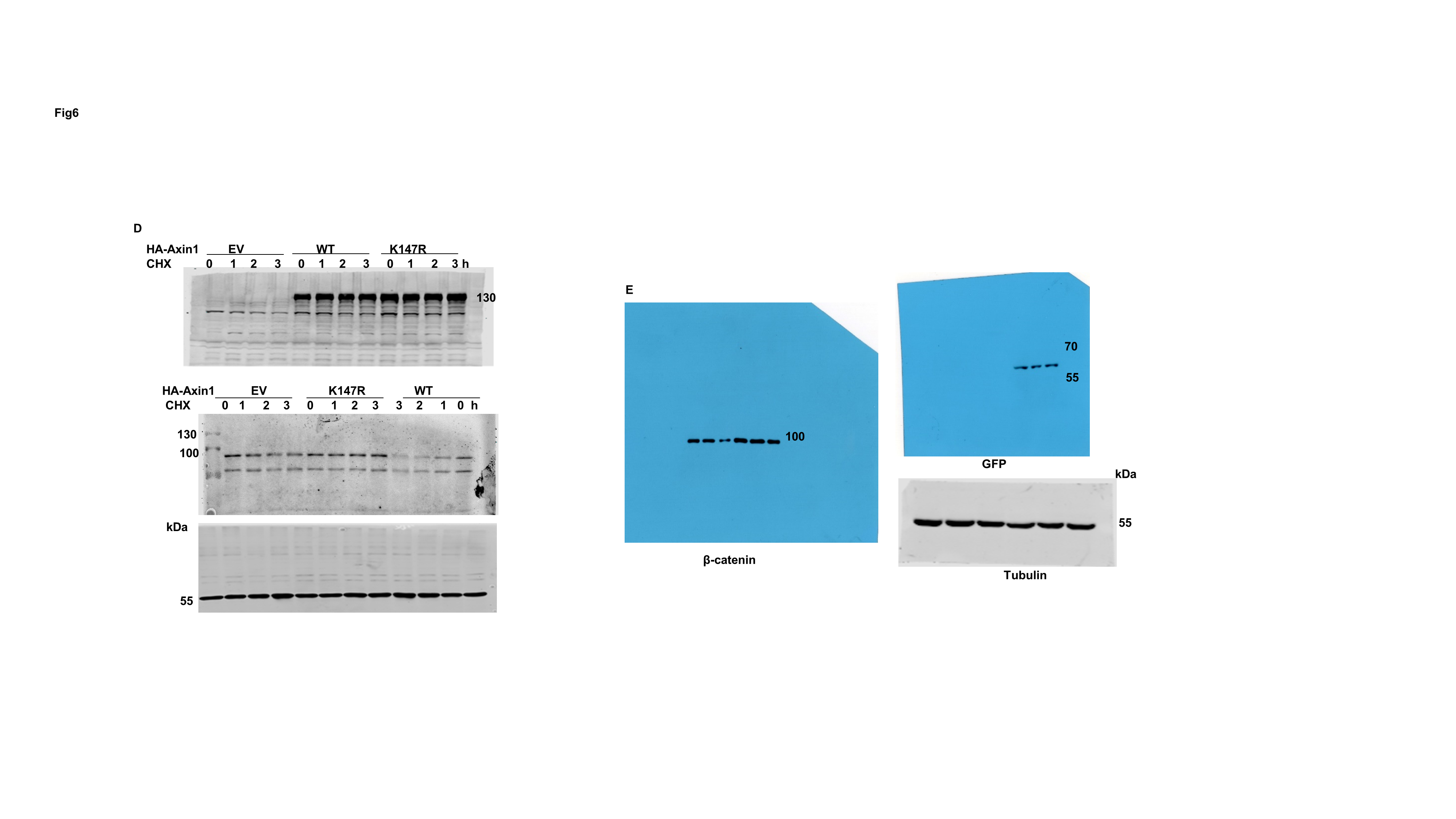

Supplement: Supplementary file 1 [file DataSheet_1.zip › 2022-5-1/幻灯片11.TIF]

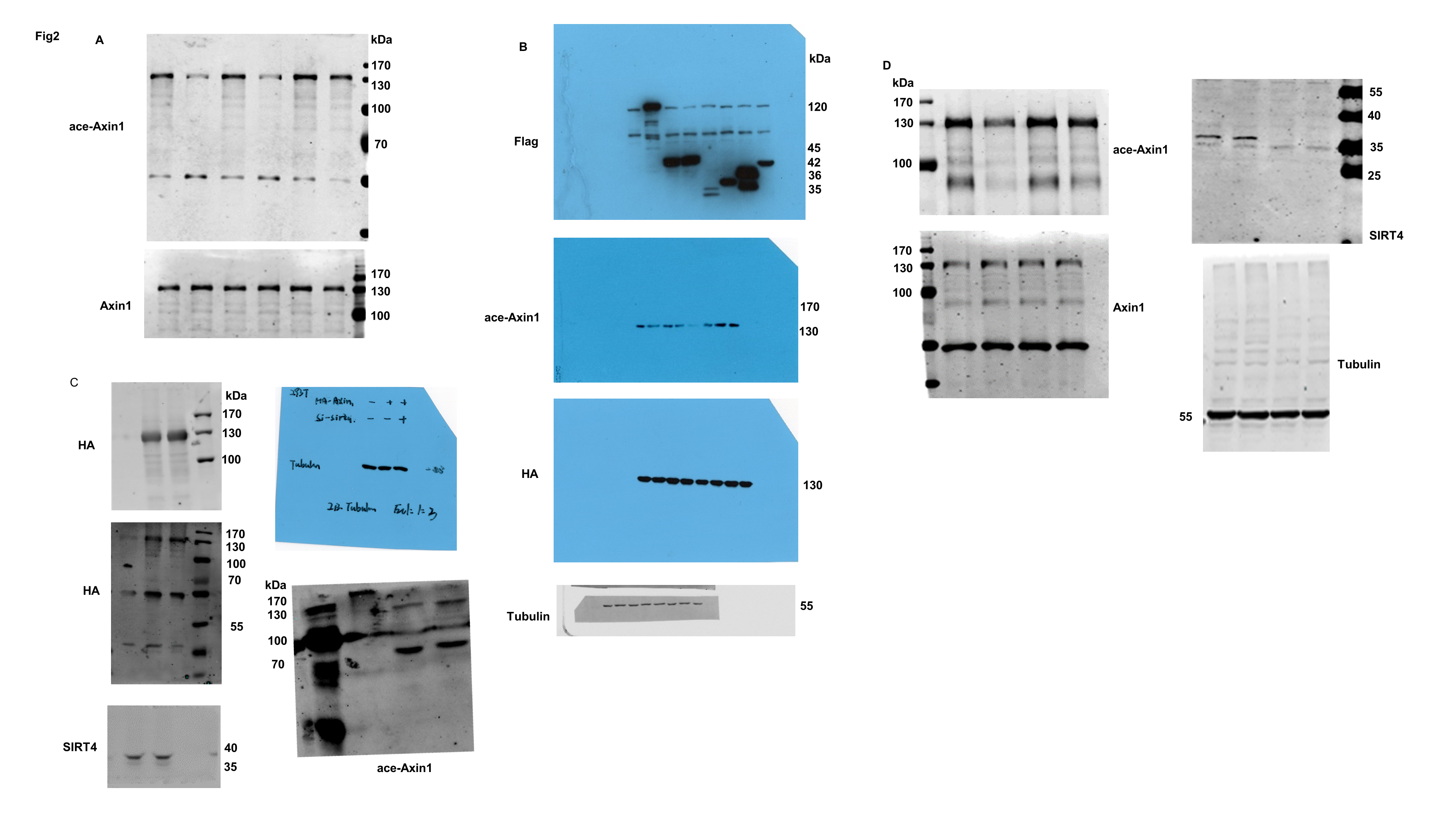

Supplement: Supplementary file 1 [file DataSheet_1.zip › 2022-5-1/幻灯片2.TIF]

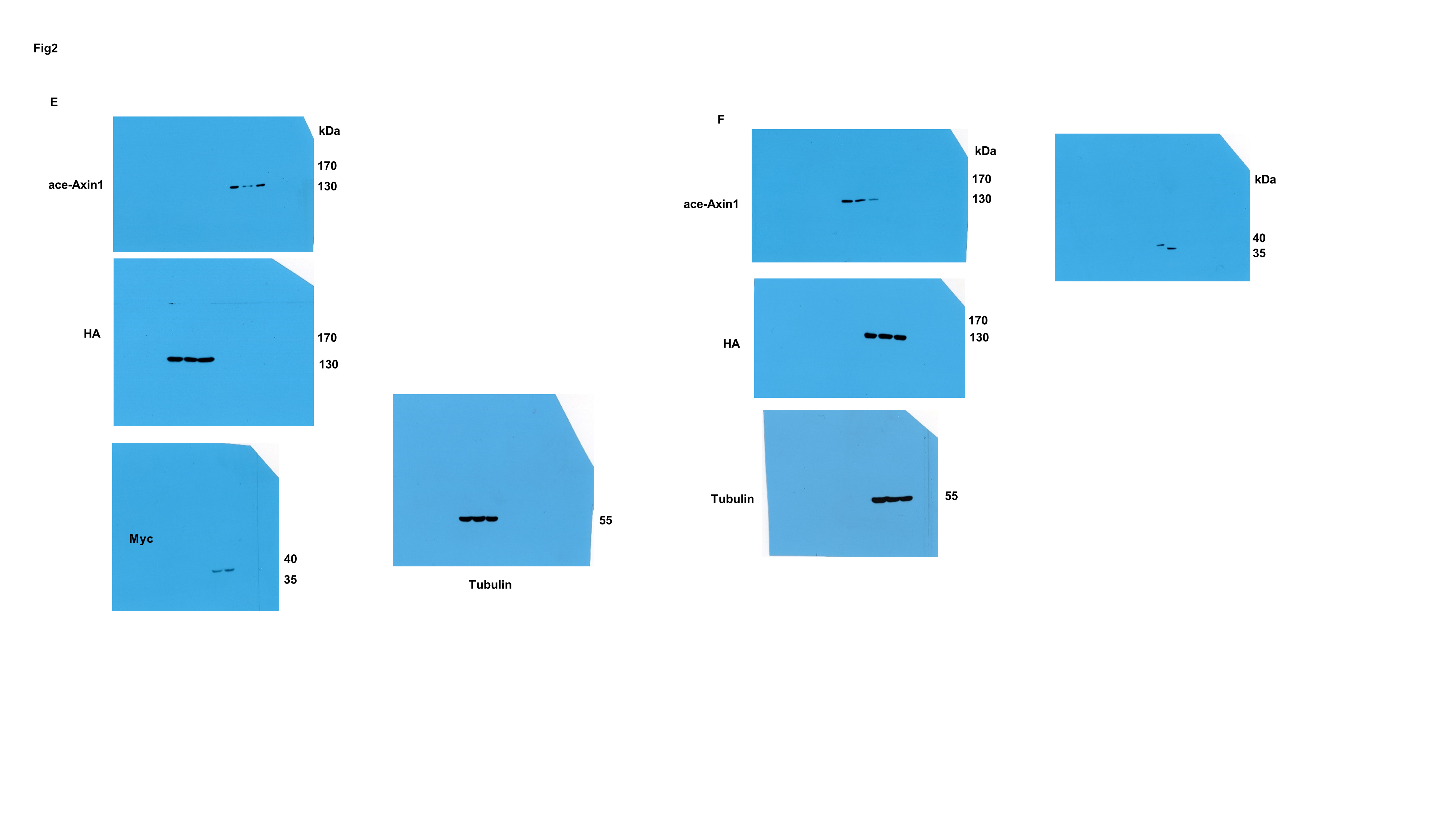

Supplement: Supplementary file 1 [file DataSheet_1.zip › 2022-5-1/幻灯片3.TIF]

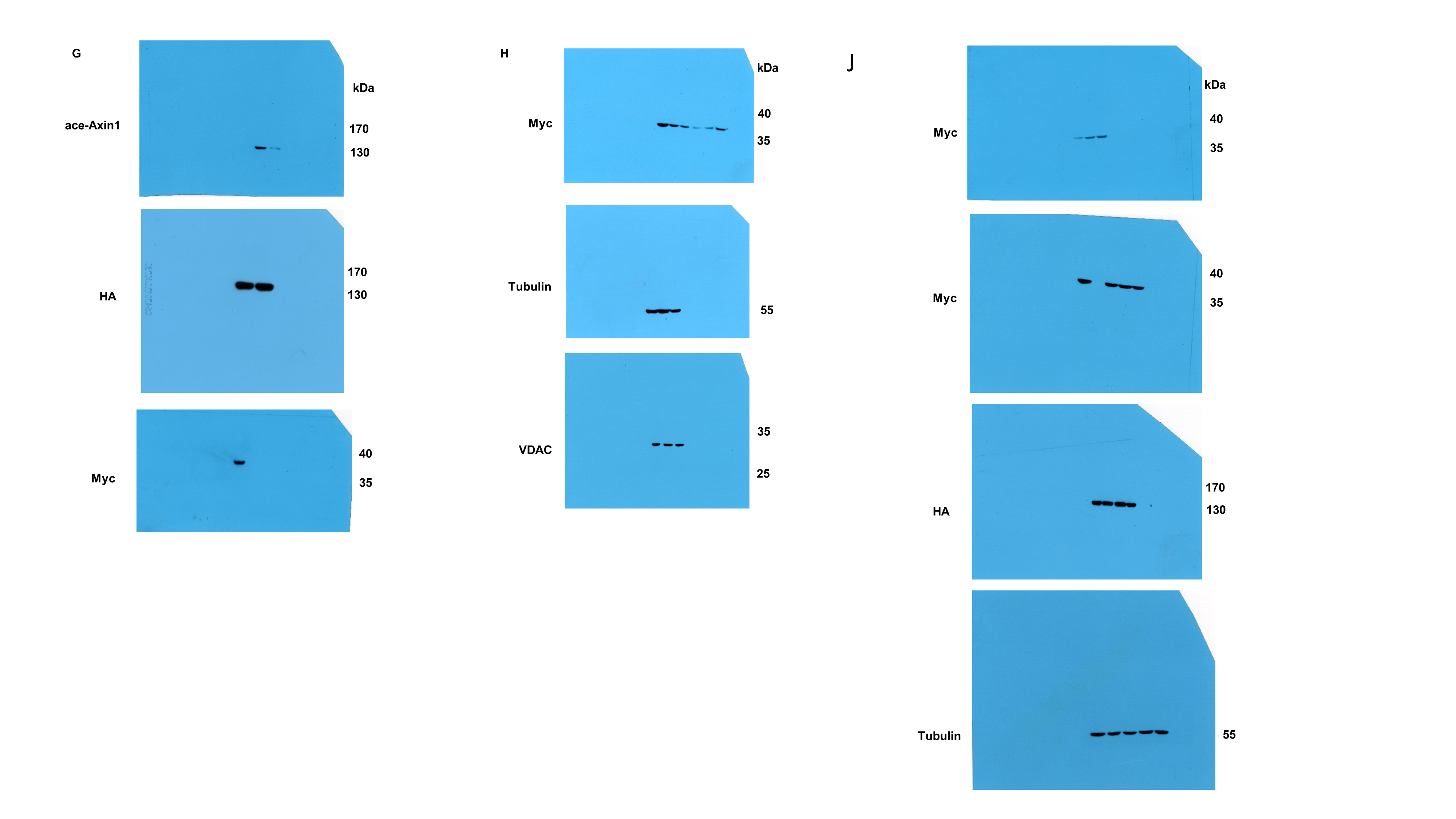

Supplement: Supplementary file 1 [file DataSheet_1.zip › 2022-5-1/幻灯片4.TIF]

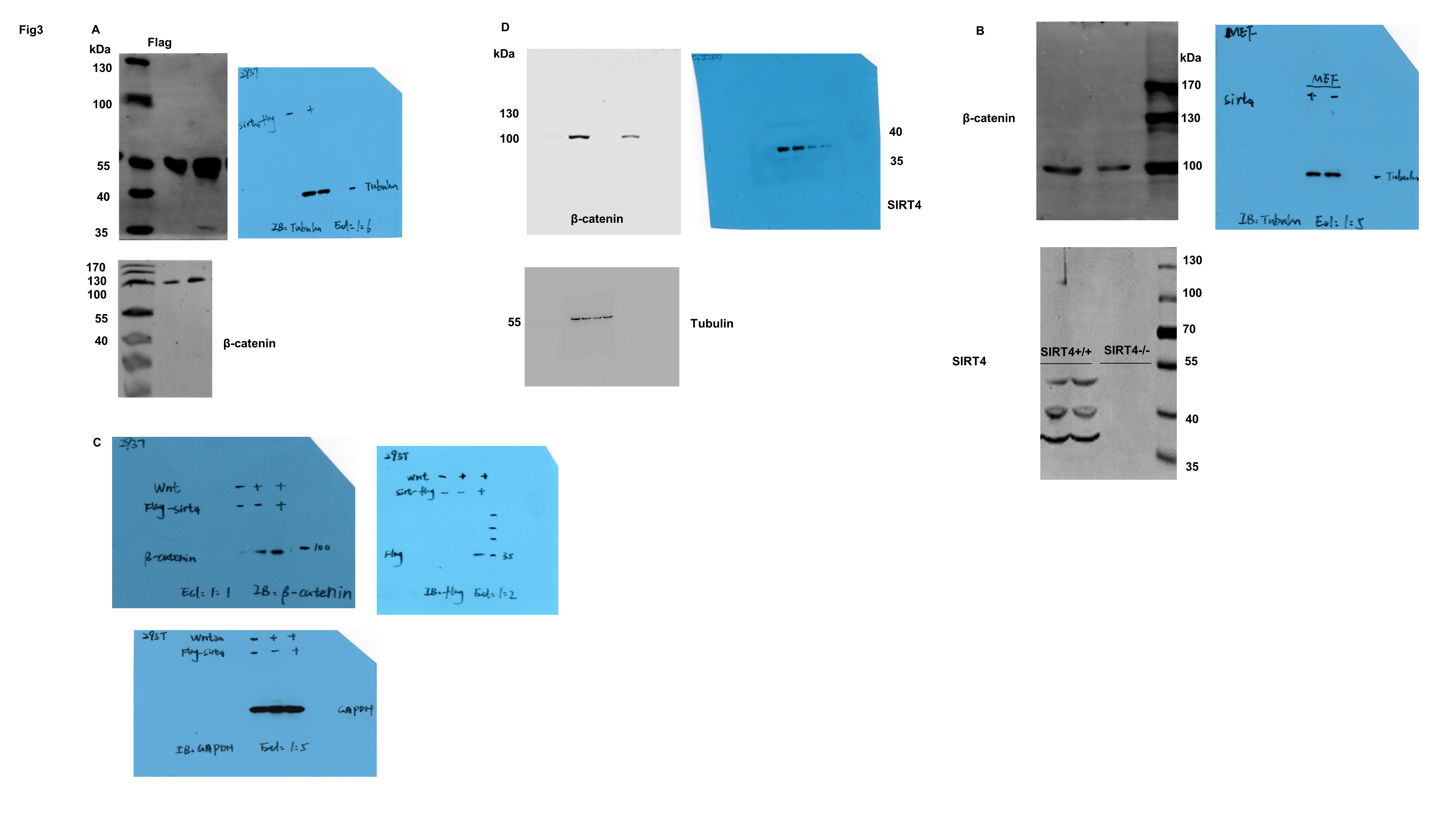

Supplement: Supplementary file 1 [file DataSheet_1.zip › 2022-5-1/幻灯片5.TIF]

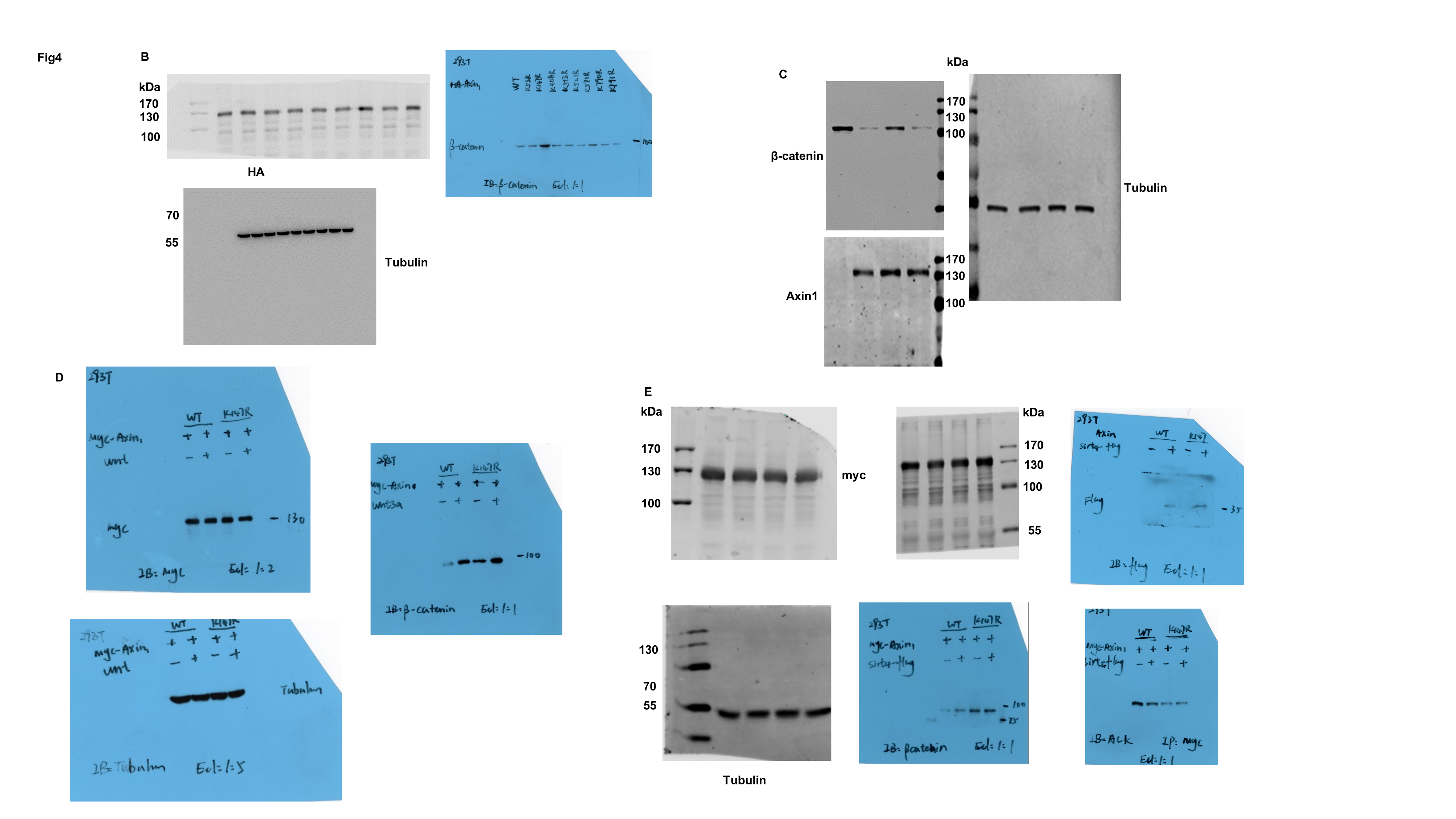

Supplement: Supplementary file 1 [file DataSheet_1.zip › 2022-5-1/幻灯片6.TIF]

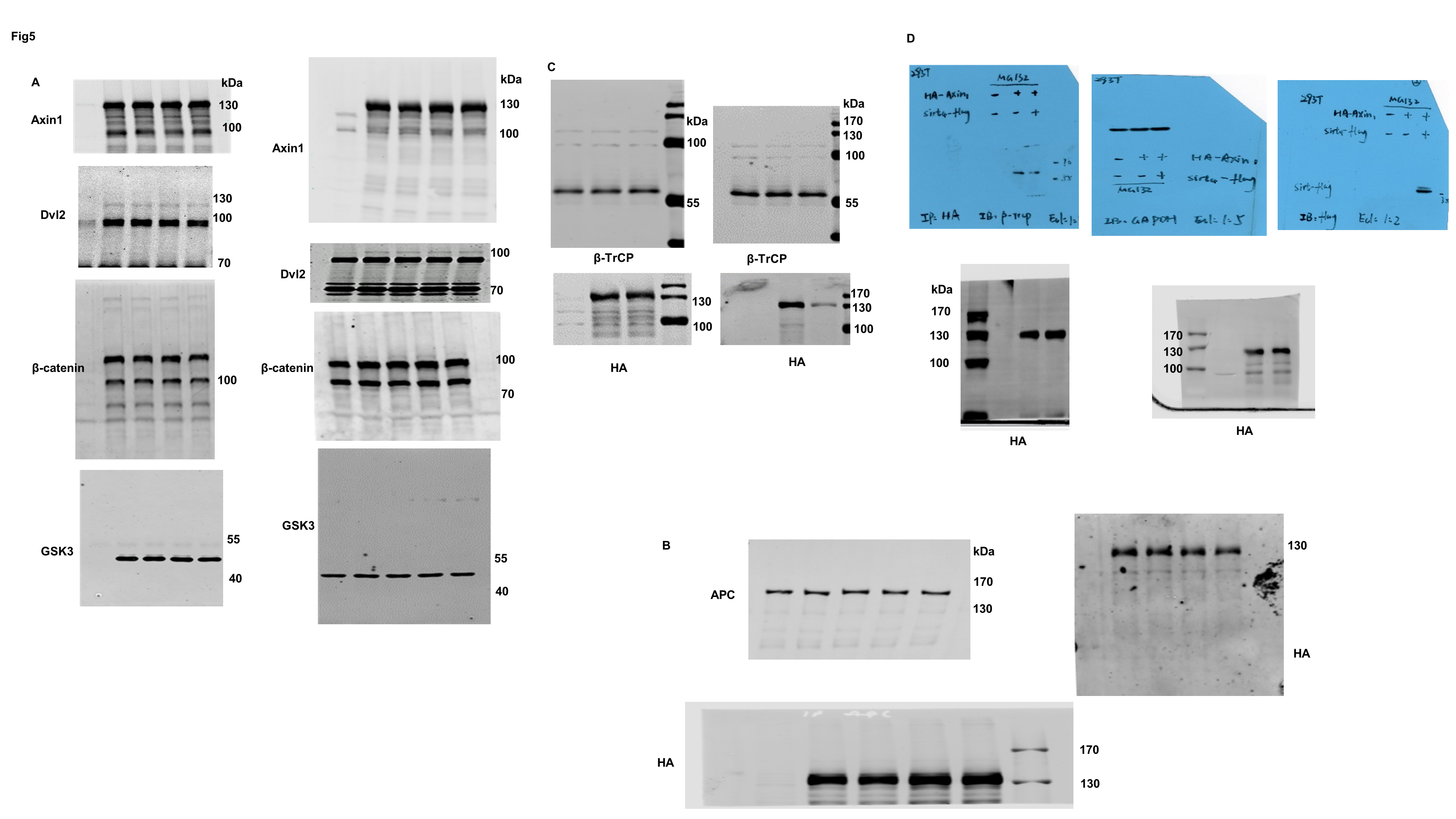

Supplement: Supplementary file 1 [file DataSheet_1.zip › 2022-5-1/幻灯片7.TIF]

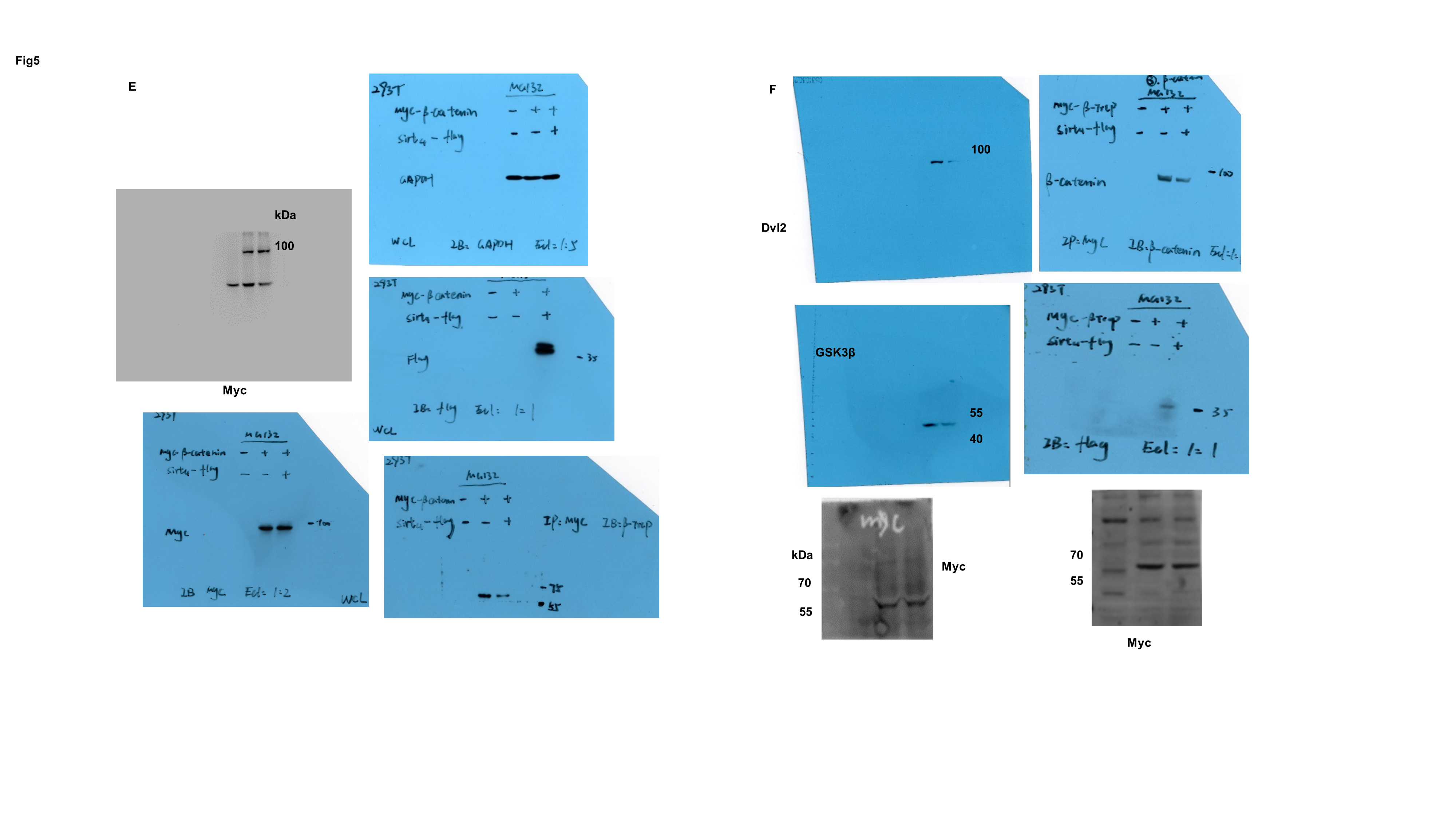

Supplement: Supplementary file 1 [file DataSheet_1.zip › 2022-5-1/幻灯片8.TIF]

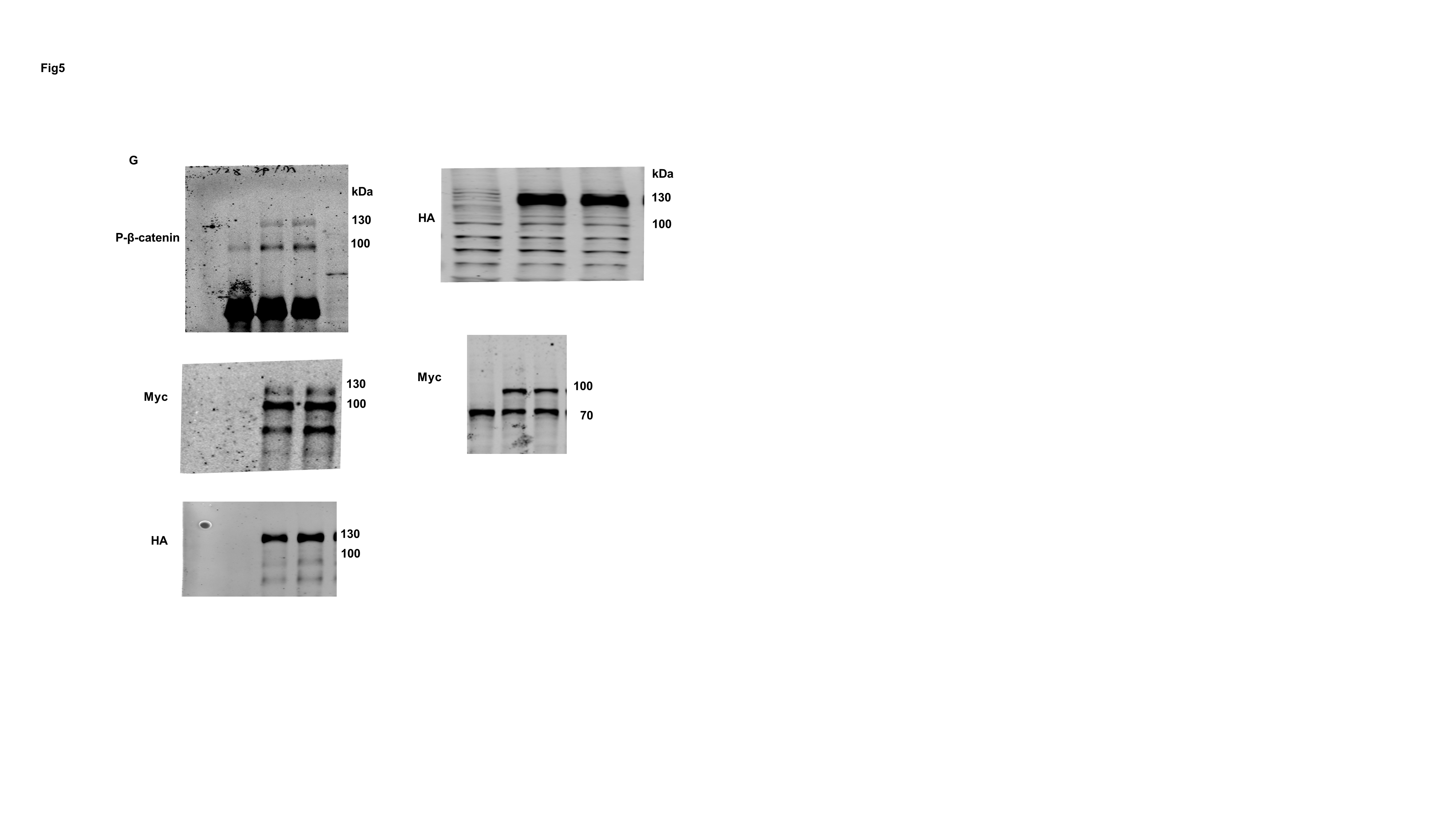

Supplement: Supplementary file 1 [file DataSheet_1.zip › 2022-5-1/幻灯片9.TIF]
